# Validity Verification of Human Pose-Tracking Algorithms for Gait Analysis Capability

**DOI:** 10.3390/s24082516

**Published:** 2024-04-14

**Authors:** Tadamitsu Matsuda, Yuji Fujino, Hitoshi Makabe, Tomoyuki Morisawa, Tetsuya Takahashi, Kei Kakegawa, Takanari Matsumoto, Takehiko Kiyohara, Yasuo Torimoto, Masaki Miwa, Toshiyuki Fujiwara, Hiroyuki Daida

**Affiliations:** 1 Department of Physical Therapy, Faculty of Health Science, Juntendo University, Tokyo 113-0033, Japan; 2 Global Development Center, Development Department, Development Section, IMASEN Electric Industrial Co., Ltd., Nagoya-shi 484-0083, Japan; 3 Department of Rehabilitation Medicine, Juntendo University Graduate School of Medicine, Tokyo 113-8421, Japan

**Keywords:** motion analysis, gait, validation, human pose estimation

## Abstract

Two-dimensional (2D) clinical gait analysis systems are more affordable and portable than contemporary three-dimensional (3D) clinical models. Using the Vicon 3D motion capture system as the standard, we evaluated the internal statistics of the Imasen and open-source OpenPose gait measurement systems, both designed for 2D input, to validate their output based on the similarity of results and the legitimacy of their inner statistical processes. We measured time factors, distance factors, and joint angles of the hip and knee joints in the sagittal plane while varying speeds and gaits during level walking in three in-person walking experiments under normal, maximum-speed, and tandem scenarios. The intraclass correlation coefficients of the 2D models were greater than 0.769 for all gait parameters compared with those of Vicon, except for some knee joint angles. The relative agreement was excellent for the time–distance gait parameter and moderate-to-excellent for each gait motion contraction range, except for hip joint angles. The time–distance gait parameter was high for Cronbach’s alpha coefficients of 0.899–0.993 but low for 0.298–0.971. Correlation coefficients were greater than 0.571 for time–distance gait parameters but lower for joint angle parameters, particularly hip joint angles. Our study elucidates areas in which to improve 2D models for their widespread clinical application.

## 1. Introduction

As people age, changes in joint range of motion (ROM) during walking occur, and gait (ambulatory) irregularities become more pronounced, especially with occurrences of osteoarthritis, stroke, and Parkinson’s disease [1]. Hence, the ability to clinically analyze patients’ simple movements during rehabilitation aids in ensuring optimal healthcare and well-being. Several quantitative examinations exist [2] that take several forms, such as timed [3] and shuttle [4] walking. A typical clinical gait assessment involves having a patient walk a distance of 10 m or less while measuring several visible parameters such as step length, step width, gait velocity, number of steps, cadence, gait cycle time, step duration, and kinematic joint angle. To achieve reliable quantitative assessments, clinicians take video recordings of these tests and apply a mixture of machine learning and manual annotations. However, owing to the high cost, complexity, and human expertise required to operate these systems, this level of service is inaccessible to most patients. Those who do participate must endure repetitive trips to specialized laboratories, the placement of dozens of markers on specific anatomical landmarks, and overly repetitious imagery sessions in which they must repeat walking tests [5,6]. Marking increases the burden on the patient, and misalignment of the markers may lead to inaccurate measurements. Additionally, the markers may alter the patient’s gait movements, making it difficult to accurately capture their normal gait.

State-of-the-art clinical systems include Vicon (Vicon Nexus2; Vicon Motion Systems Ltd., Oxford, UK) and Optitrak [7,8]; Vicon is the gold standard, meaning that it produces the most accurate and consistent results, and its internal statistical processes are known to be valid. “Internal validity” is essentially a guarantee that the processes used to make quantitative assessments are sound, repeatable, and confident at a very high level (e.g., 95–98%, depending on the measure). For Vicon, this guarantee refers to its function of making predictions based on three-dimensional (3D) video input. Therefore, it not only handles stereoscopic image data well but also keeps track of features across the sequential frames of any given video. However, Vicon requires anatomical markers to be placed on the patient.

Recently, markerless systems that use accelerometers or other on-body data collection and transmission units have been developed; however, they are even more difficult to administer than anatomical marker solutions. Moreover, they are notoriously buggy when acquiring accurate kinematic data. Microsoft’s Kinect motion-sensing devices were a major markerless breakthrough in 2010, and they are easy to operate, easily portable, and highly economical. As such, several gait-related studies have reported significant differences between the results obtained from Kinect gait measurement solutions and the baseline Vicon system. To understand why, researchers performed an internal validity analysis of the Kinect device [9] by comparing the statistical rigor and parametric continuity of its evaluation of an 8 m walking test with that obtained with the Vicon system. Based on its intraclass correlation coefficients (ICCs), the internal validity of Kinect could only be guaranteed at very low data acquisition rates, which renders it unsuitable for clinical use.

OpenPose is an open-source (i.e., free) convolutional neural network that was created primarily for object (e.g., face) identification and position (e.g., posture) recognition. It also intakes video frames but only one at a time. Additionally, it was not developed to deal with 3D binocular image pairing [7,10]. OpenPose does not require markers or accelerometers. Studies have demonstrated its potential for clinical gait recognition applications [5,11]. However, it is more limited than Kinect owing to its inability to remember features across video frames. Nevertheless, OpenPose holds great promise. Hence, we believe that if it can be properly adapted, OpenPose will provide a simple, inexpensive, and valid motion analysis system that can extend necessary treatments to patients worldwide.

Existing Vicon and OpenPose comparison studies are based on squatting [1] and treadmill walking [5], both inferring high reliability and validity. Recent studies on joint position recognition during walking suggest that the differences observed in the corresponding joint positions were minor [11,12]. Such comparisons should also consider different joint ROM settings. Notwithstanding a previous fixed-camera study [5], the reliability and validity of gait recognition using OpenPose for walking on level ground has not been completely investigated.

The aim of our study was to clarify and assess the internal validity of OpenPose based on the Vicon gold standard. We limited the scope of this assessment to measuring the time and distance factors and joint angles of the hip and knee joints in the sagittal plane with varying speeds and gaits during level walking (maximum and tandem). To obtain 3D images, we linked two Imasen 2D cameras and combined their inputs. We expected that this configuration would overcome the limitations of using OpenPose to evaluate video from a single, fixed-point camera. To demonstrate the efficacy of gait analysis using OpenPose, we compared the performance of the Imasen system, which employs skeletal estimation through OpenPose for gait analysis, with that of the Vicon system. The Imasen system uses OpenPose for skeletal estimation on camera images in both the sagittal and frontal planes, computing parameters such as time and distance during walking, along with joint angles of the hip and knee joints in the sagittal plane. This system offers clear advantages over the Vicon system in terms of cost-effectiveness and simplicity.

## 2. Materials and Methods

### 2.1. Participants

Previous studies targeting healthy people in this type of internal validity study indicated that at least 20 participants were required [6,9,13]. Therefore, 20 healthy young participants (4 males and 16 females, mean age: 20.5 ± 2.5 years, mean height: 160.9 ± 6.8 cm, mean mass: 51.0 ± 6.4 kg) were recruited. Individuals with a history of serious injuries, such as ligament or musculoskeletal injuries, neurological disorders, and fractures, were excluded. This study was approved by the Ethics Committee of Juntendo University (#20-004), and all participants provided informed consent

### 2.2. Motion Task

Participants were randomly requested to perform flat-floor, comfortable, maximum-speed, and tandem walking trials. Videos of at least 10 trials of three gait cycles (based on patient stability and fatigue) were recorded.

### 2.3. Data Collection and Processing

**Figure 1:**
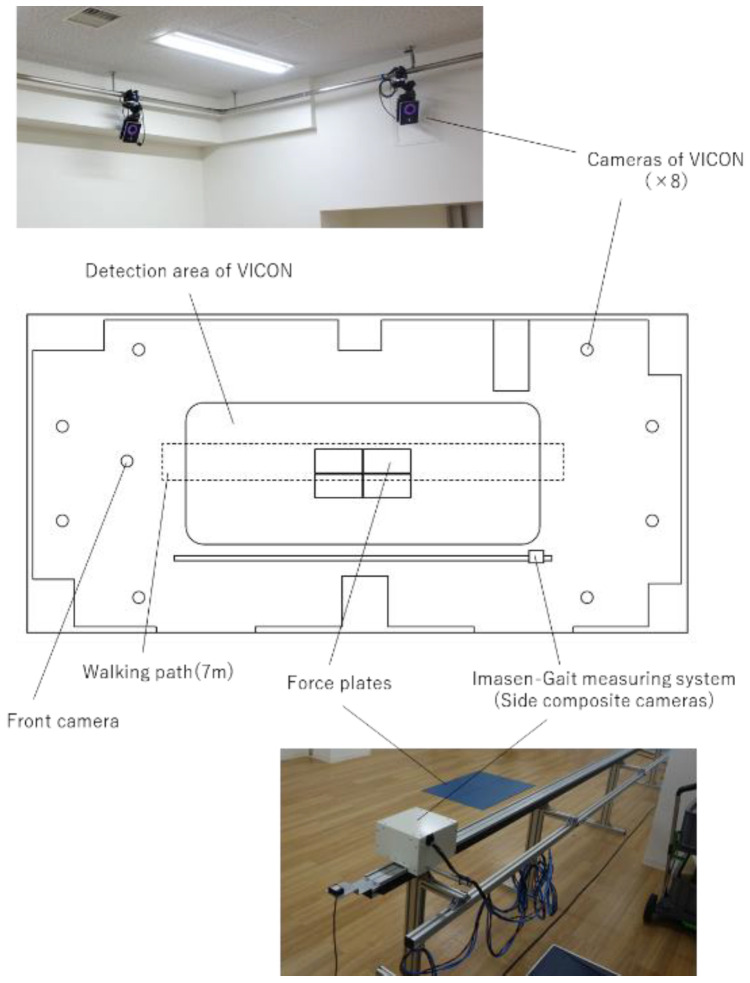
Vicon gait measurement environment.

**Figure 2:**
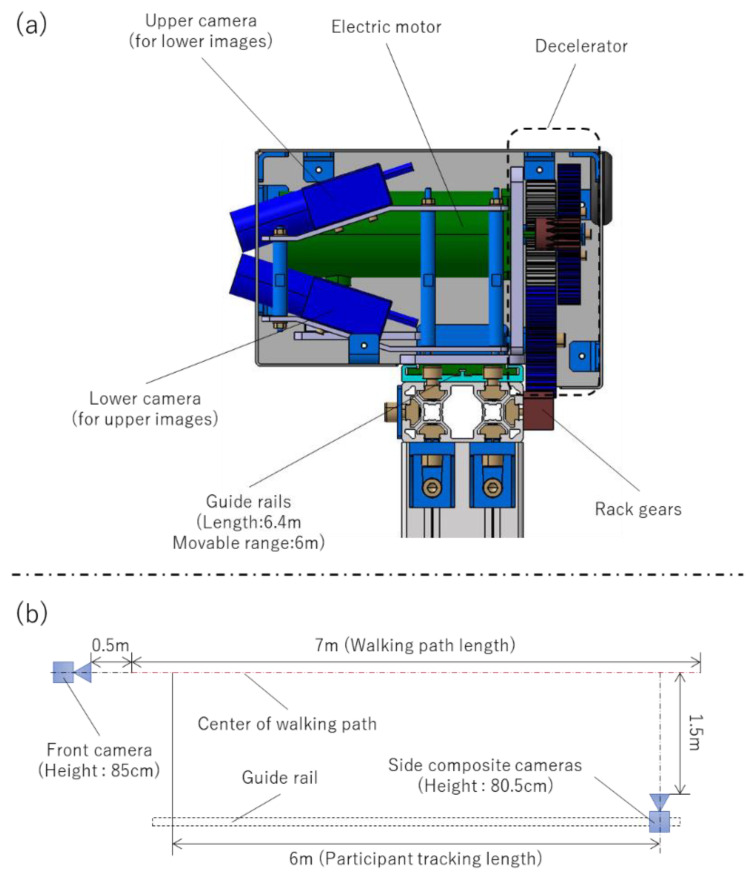
Imasen and OpenPose measurement environment. (**a**) Camera structure. (**b**) Location of walking paths and cameras.

An eight-camera 3D Vicon configuration (Vantage 5 Camera [5 MP resolution, 500 megapixels]) was used to acquire baseline internal parametric statistics (Figure 1). A three-camera 2D Imasen configuration––a paired set for 3D rendering and a single camera set orthogonal to the pair (Figure 2)––was also used to simultaneously record the trials. The paired camera system was set to the side of the walking path on a motorized guide rail at a center-of-lens height of 80.5 cm from the walking surface facing the patient. Beginning at a position of 1.5 m in front of the participant, the camera system, situated on the side rail, tracked along the participant for up to 6 m to capture constant-distance kinematics. The OpenPose algorithm was then used to process the data obtained from the Imasen cameras.

### 2.4. Data Analysis

Peak angles of hip flexion and extension, knee flexion and extension, and trailing limb angle (TLA) were measured for four successful gait cycles, including the ROM of each. Heel contact was identified by software analysis of the images in each frame to establish the gait cycle.

According to the Vicon body marker placement protocol, 39 reflective markers were attached bilaterally to the anterior superior iliac spine (ASIS), posterior superior iliac spine (PSIS), lateral thigh, lateral femoral epicondyle, lateral shank, lateral malleolus, second metatarsal head, and calcaneus. The pelvic segment coordinate system was defined using ASIS and PSIS markers, where the pelvic origin was taken as the midpoint of both ASIS markers, from which the *y*-axis (left-to-right) was established. The *x*-axis started from the midpoint of both PSIS markers to the midpoint of both ASIS markers and continued forward. This direction was adapted in all segments.

Vicon’s Clinical Manager software (VICON Nexus 2, Vicon Motion Systems, Oxford, UK) was used to calculate the pelvic angle in the global coordinate system and the relative angles between the other coordinate systems using Euler angles. The angles of pelvic elevation and depression were measured between the transverse axis in the frontal plane of the laboratory (i.e., the horizontal axis perpendicular to the participant’s axis of progression) and pelvic *y*-axis, which represented hip flexion and extension, knee flexion and extension, ankle dorsiflexion, and plantar flexion.

**Figure 3:**
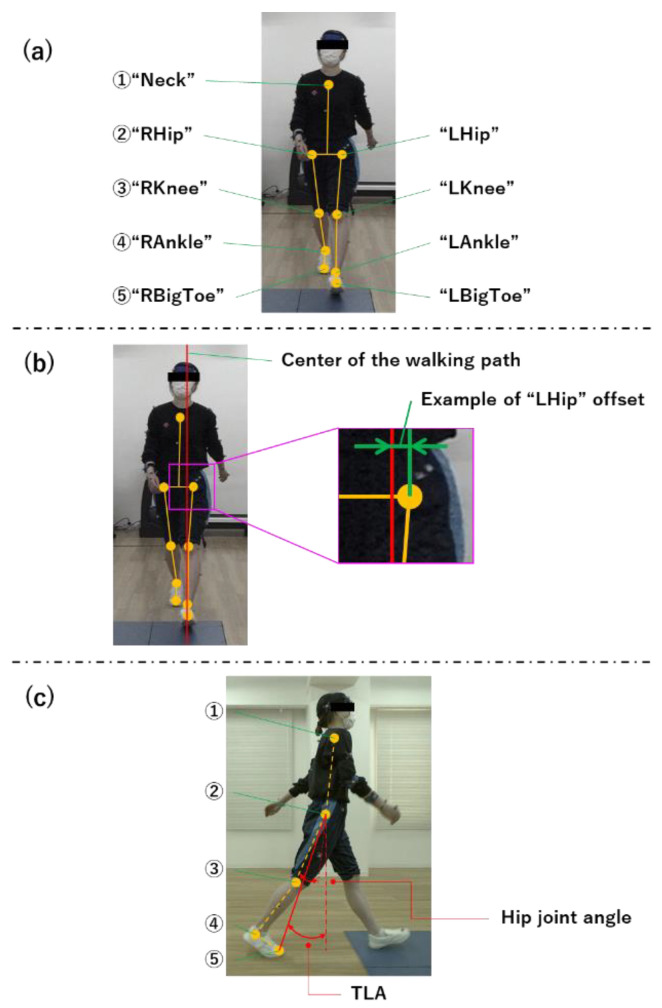
OpenPose nodes. (**a**) Nodes at each site on the skeletal estimate. (**b**) Center of walking path and offset. We converted each coordinate of the front camera created to coordinates in real space, calculated the value of offset from the center of the walking path with respect to those coordinates in real space, and calculated the average of those offset values. (**c**) How to calculate the TLA. TLA, trailing limb angle.

**Figure 4:**
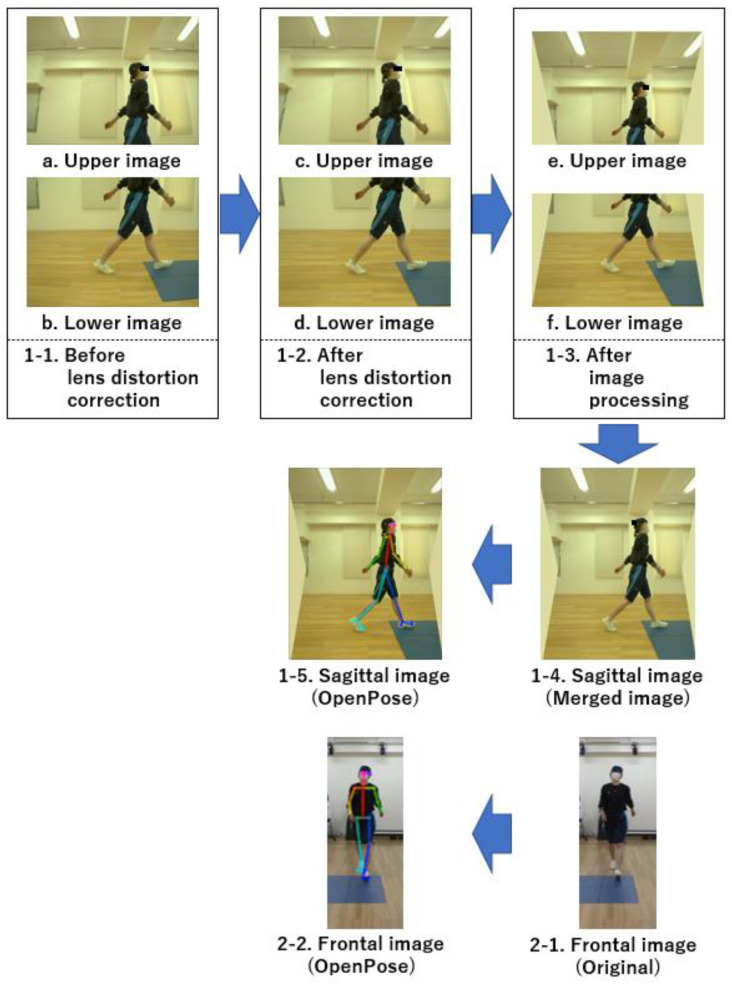
Flow of image processing up to skeletal estimation.

**Figure 5:**
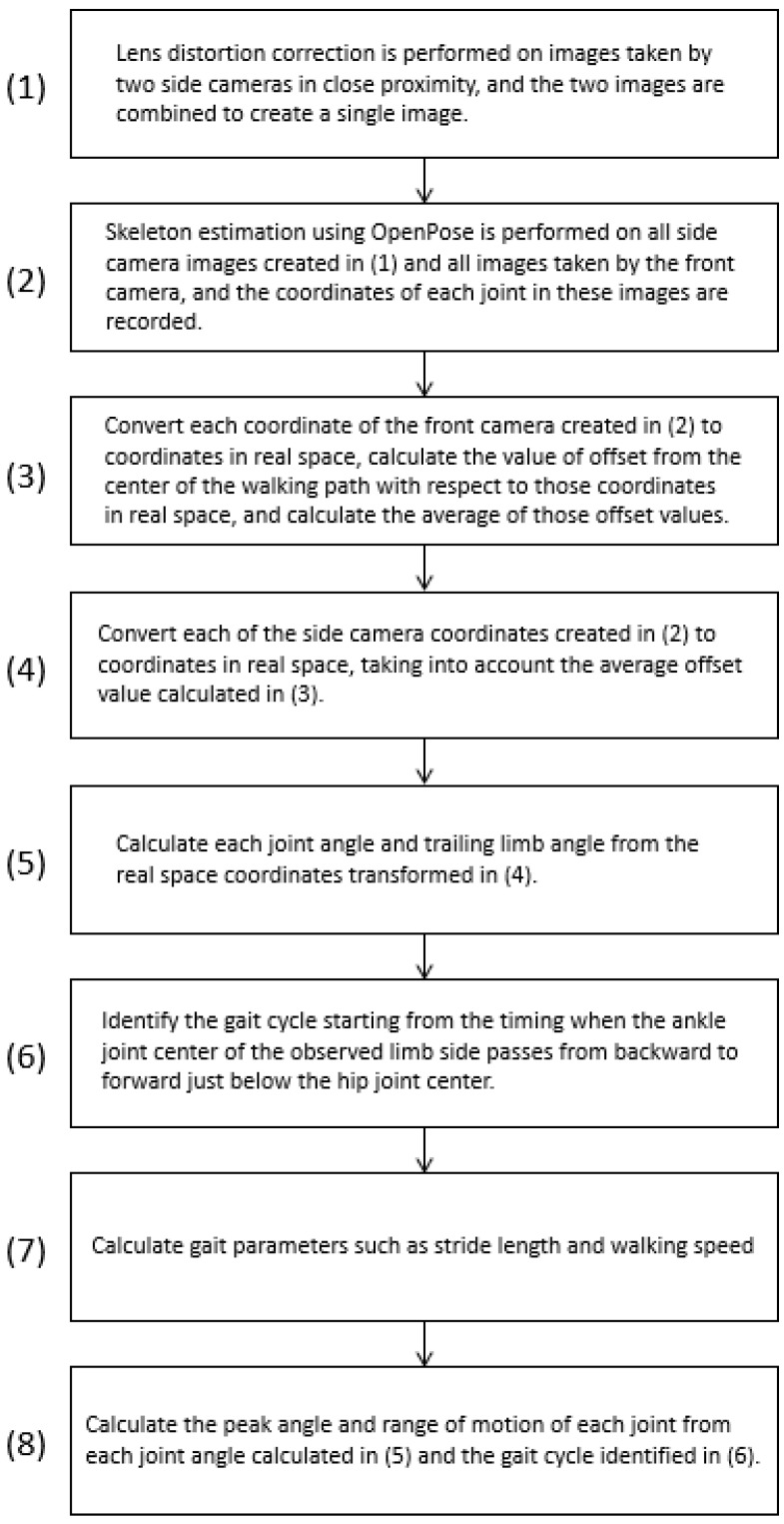
OpenPose walk and gait parameter outputs. TLA, trailing limb angle.

With respect to OpenPose, the segment and joint angles were measured from the estimated feature points of each joint, placement and definition of each feature point, and segment and joint angles between each feature point measured from the obtained marker coordinates (Figure 3, Figure 4 and Figure 5).

This study involved measuring various gait parameters, peak angles of hip and knee joint extension and flexion, and TLA. The ROM during each gait cycle was calculated. The starting point of the gait cycle was defined as the time when the ankle joint center of the observed limb in the swing phase passed from backward to forward just below the hip joint center in the sagittal plane. As both systems detected multiple gait cycles per trial, this study identified the same gait cycle based on the position of the floor reaction force meter linked to the Vicon system for comparative verification.

The calculation method for each gait parameter and joint angle during one trial with the Imasen system is outlined as follows:

1. Lens distortion in images captured by the side camera (sagittal plane) was corrected, and images from the two cameras were combined.
2. Skeletal estimation using OpenPose was performed on images from both the side and front cameras, recording the coordinates of each joint node.
3. Joint node coordinate information in each frame of the front camera was converted to real space coordinates, considering height information and joint node coordinates.
4. Joint node coordinate information in each frame of the side camera was converted to real space coordinates, accounting for the distance between the side camera and the walking path center plane.
5. Each joint angle and TLA for each frame was calculated, considering the angles between specific joint nodes in the sagittal plane.
6. The gait cycle was identified based on the timing of the ankle joint center passing directly under the hip joint center.
7. Each gait parameter, including stride time, stride length, and gait speed, was calculated.
8. Peak angles and ROMs for each joint during the identified gait cycle were calculated.

Processes (1) to (2) were performed using a camera control program written in C++, whereas (3) to (8) were automatically processed using a MATLAB program specifically designed for this purpose. The output for the results of each trial was a CSV file. Additionally, a MATLAB program was created to compare the output of Vicon and the Imasen systems for the same walking cycle, extracting numerical values from the log file of each trial for comparison and verification. Regarding the flow of image processing up to skeletal estimation (Figure 4), in both processes, OpenCV functions were used. Lens distortion was corrected (1–2) using lens distortion correction data created in advance using the chessboard (using OpenCV’s calibrateCamera function). Further, the image was processed (1–3) with the projection plane at 1.5 m (distance between the side compound camera and the center of the walking path) and projectively transformed according to elevation and tilt angles (adjusting transformation parameters on a wall with a pre-drawn grid and using OpenCV’s warpPerspective function with those parameters). The upper and lower images projectively transformed in (1) to (3) were combined into a sagittal plane image (1–4) using predefined joint coordinates (using OpenCV’s matchTemplate function). A series of adjustments were made after fixing the camera on a highly rigid pedestal, avoiding the need to repeat them every time the system was moved or set up.

### 2.5. Statistical Analysis

ICCs and 95% confidence intervals (CIs) were used to calculate test–retest, intra-rater, and inter-rater reliabilities. To confirm whether the data obtained by OpenPose agreed with those of Vicon, the ICCs between their data were calculated. ICCs less than 0.5 indicated poor agreement, those between 0.5 and 0.75 indicated moderate agreement, those between 0.75 and 0.9 indicated good agreement, and those greater than 0.90 indicated excellent agreement [14].

Linear regression was performed using the data obtained with OpenPose and those obtained with Vicon. The correlation coefficients (r) were determined, and values between 0.1 and 0.3 indicated small correlations, those between 0.3 and 0.5 indicated medium correlations, and those greater than zero indicated large correlations [15]. Therefore, the coefficients of determination (R^2^) between 0.01 and 0.09 indicated small correlations, those between 0.09 and 0.25 indicated medium correlations, and those greater than 0.25 indicated large correlations. For Vicon vs. Imasen, the internal consistency of each gait parameter was determined using Cronbach’s alpha, which represented the extent to which the gait parameters measured similar constructs. Coefficients greater than or equal to 0.7 were considered acceptable. Alternatively, for Vicon vs. OpenPose, the agreement between the data was assessed using Bland–Altman analysis. All statistical analyses were performed using IBM SPSS v.24.0 (IBM, Tokyo, Japan), and the significance level was set at *p* value < 0.05.

## 3. Results

### 3.1. Data Acquisition Rate

For Vicon, we found that some gait data were missing due to trials in which it was difficult to visually acquire markers. For Imasen, there were deficiencies with the cameras. The data acquisition success rates were 94.0% for Vicon and 93.5% for Imasen. The two gait measurement systems did not comprehensively analyze all aspects of gait. For instance, Vicon encountered challenges in analyzing certain practices owing to missing reflex markers. Similarly, the Imasen system involved difficulties in analyzing some cases owing to challenges in integrating gait videos. The instances of successful analysis were documented.

### 3.2. Test–Retest and Intra-Rater Reliability Analysis

**Figure 6:**
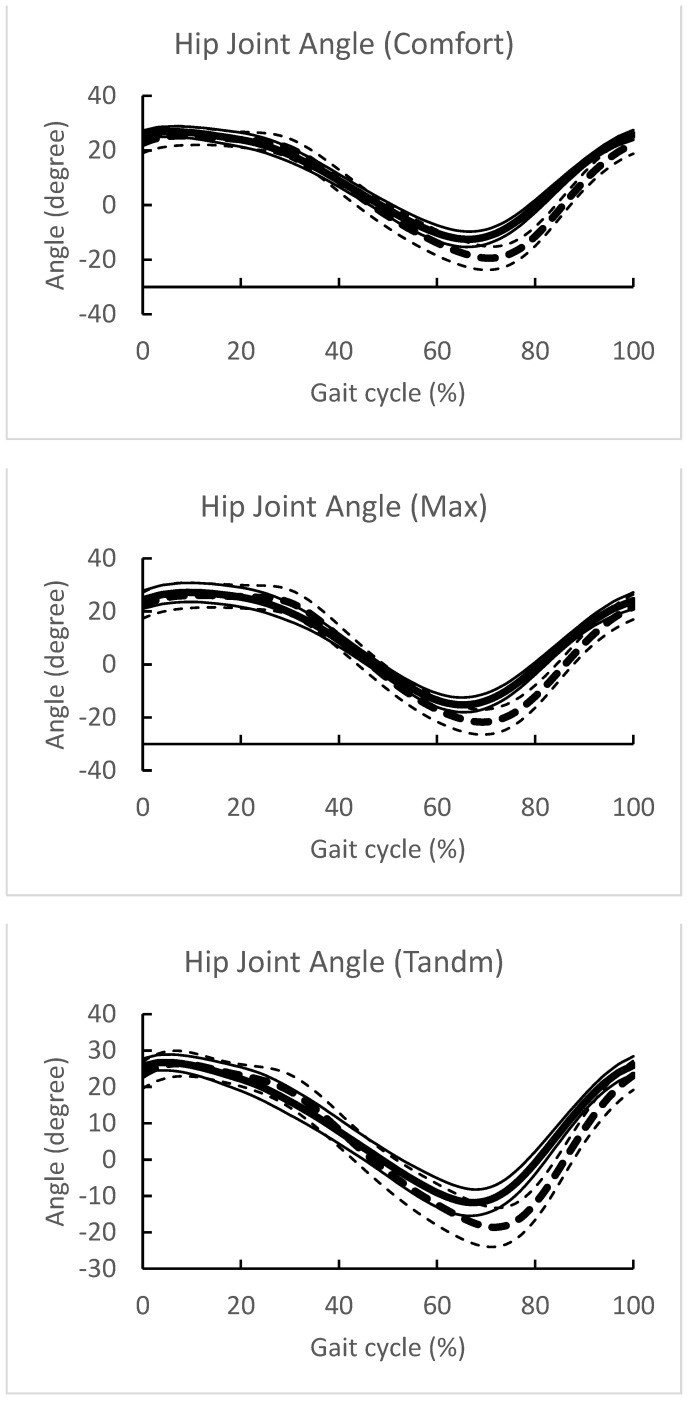
Hip joint angle change in one gait cycle. The changes in joint angles for each of the three gait patterns are summarized. The identification of one gait cycle was normalized, so that 0 was the time when the central axis of the hip joint crossed over the central axis of the ankle joint, and 100% of one gait cycle was identified. The solid line indicates the mean value for all subjects in the Imasen gait system, and the dashed line indicates the mean value for all subjects in the VICON system. The SD of the SD is also described.

**Figure 7:**
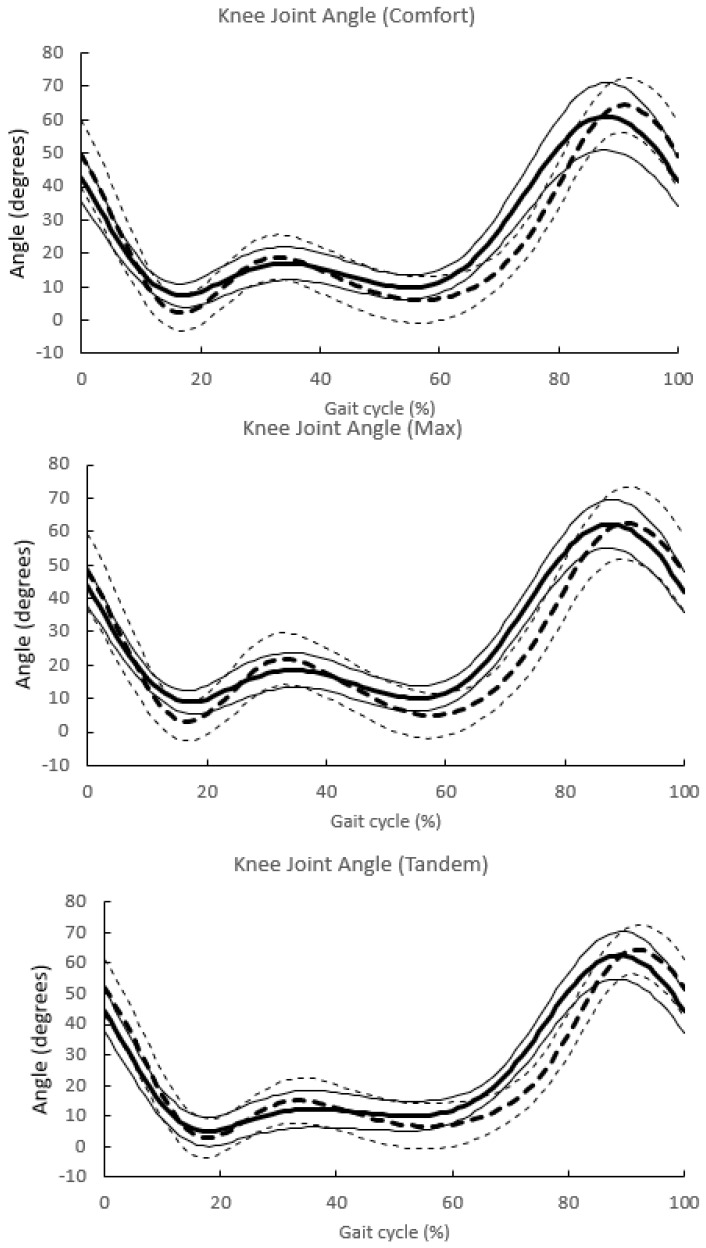
Knee joint angle change in one gait cycle.

Figure 6 and Figure 7 show the angular changes of the hip and knee joints, respectively, during one gait cycle in the three gait patterns obtained with the Vicon and Imasen systems.

- The changes in joint angles for each of the three gait patterns are summarized.
- The identification of one gait cycle was normalized, so that 0 was the time when the central axis of the hip joint crossed over the central axis of the ankle joint, and 100% of one gait cycle was identified.
- The solid line indicates the mean value for all subjects in the Imasen gait system, and the dashed line indicates the mean value for all subjects in the VICON system. The SD of the SD is also described.

Table 1 and Table 2 present a comparison of the Imasen and Vicon systems, with the ICCs and mean and standard deviation (SD) for the gait parameters during three sessions of overground walking. For Vicon, the ICC values were greater than 0.729 for all gait parameters. With the Imasen system, the ICC was moderate at 0.428 when walking at the maximum speed but high in most other cases. From the test–retest reliability analysis, all items were found to have good or excellent ICC values (Table 1 and Table 2).

### 3.3. Criterion Validity

The ICCs ((2, k) or (3, k)), R^2^, and Cronbach’s alphas for Vicon and Imasen are listed in Table 3 and Table 4, respectively. The relative agreement for the two systems was excellent for the time–distance gait parameter for each gait condition (ICC: 0.866–0.994, *p* < 0.001) and moderate to excellent at each gait motion contraction range (ICC: 0.521–0.971, *p* < 0.05), except for the hip joint flexion angle (maximum gait speed: ICC = 0.298–0.372, *p* > 0.05).

Regarding the internal consistencies of the gait parameters of each gait condition, the time–distance parameters were good for Cronbach’s alpha coefficients ranging from 0.899 to 0.993 (Table 3), but they tended to be lower for Cronbach’s alpha coefficients ranging from 0.298 to 0.971 (Table 4). The correlation coefficients (*R*^2^) were greater than 0.571 for time–distance gait parameters, but they were lower for joint angle parameters, particularly the hip joint flexion angle (*R*^2^ = −0.022–0.267).

The Bland–Altman plots for each joint angle at maximum flexion obtained from the Vicon and Imasen systems are shown in Figure 5. The *x*- and *y*-axes represent the average and difference, respectively, between the method outputs. Measurement and proportional errors were unlikely to occur for many of the items. However, the angular data were prone to error (Table 5 and Table 6).

## 4. Discussion

The ICC (1, k) indicated that the test–retest reliability between the markerless and marker-based systems nearly completely agreed. ICC (2, k) and ICC (3, k) were highly reliable with respect to distance and time factors, and we confirmed that differences between devices were unlikely to occur. The reliability and validity of the measurements were also shown to be high, with low measurement errors. We observed large coefficients of determination without proportional biases for most parameters of the hip flexion–extension ROM and knee joint. The ICCs showed good-to-excellent agreement, particularly for the TLA, during each gait condition. However, the coefficients of determination were non-significant, and the ICCs were poor, with fixed and proportional biases. The hip joint angle under each gait condition was lower than that of the knee joint angle.

There were large coefficients of determination between the data obtained by Vicon and OpenPose for the hip flexion–extension ROM and most knee parameters. Moreover, no proportional biases were observed. The ICCs were moderate to good for most ROM parameters in the sagittal plane of the lower extremity, with a 95% CI within a narrow range, particularly for the hip flexion–extension ROM during running, knee flexion angle during running, and knee joint ROM during slow walking. Therefore, the validity of OpenPose is supported for lower-limb ROM in the sagittal plane. However, we found that movements involving lateral rotation of the knee and rotation of the pelvis and trunk could not be measured with Imasen. Additionally, the reliability of the Imasen system was somewhat reduced during tandem walking. This is because OpenPose is inherently a 2D image analysis system. Hence, transverse plane rotations cannot be accurately tracked over multiple frames. However, motion tasks without transverse plane rotations offered valid results. Furthermore, there were differences in angle measurement methods between Vicon and OpenPose regarding hip joint angles due to pelvis and spine motions.

The minimal detectable change in temporal gait parameters obtained using 3D motion capture during inter-session and test–retest experiments of healthy human gaits has been reported to range from 0.02 to 0.08 s [16,17]. Several previous studies have used markerless analyses to study gait patterns of walking and other movements [1,12]. Our findings are consistent with those of previous reports as we also found that 3D markerless gait evaluation using the Imasen system provides promising quantitative information. This system is expected to enable low-cost, simplified motion analysis. Further verification of the reliability and validity of the device should be the target of future research. In addition, the system requires only a few cameras, and the accuracy has been widely verified [18]. Usually, the number of cameras and other factors also affect measurement accuracy, making it suitable for clinical use but somewhat limited for research purposes. Skeletal estimation has proven to be highly accurate and can be used clinically. However, we believe that each system should be used with the understanding that Vicon and OpenPose have different joint angle settings. Moreover, using these images for clinical evaluations (e.g., Edinburgh Visual Gait Score) [19] could potentially alleviate the therapists’ evaluation burden. Additionally, the simplified acquisition of movements could help accumulate data from many people. A recent study has shown a significant increase in running speed when students are taught running by extracting movement features from KinectV2 using a feature extraction algorithm [20]. In the future, the development of a system allowing for the simple acquisition of daily clinical data, extracting them using feature extraction algorithms and other methods, and providing feedback to the patient can guide the planning of treatment programs.

Nevertheless, the current study has some limitations. First, the Imasen system can only be used to detect movements in the sagittal plane and those of the hip and knee joints. In the future, we will consider the possibility of analyzing all joints and the anterior and horizontal planes. Second, the participants were limited to young, healthy people, implying that we have only demonstrated the validity of the 2D systems in 3D tasks based on this specific population. On the other hand, we demonstrated the ability to use these systems to recognize a variety of gaits on flat ground rather than on a treadmill, which is significant for future studies.

## 5. Conclusions

This study verified the validity of the 2D OpenPose gait analysis system for 3D gait recognition tasks in several ROM areas. For example, the hip and knee joint angles measured using OpenPose were significantly associated with the Vicon 3D system, and only fixed biases (not proportional biases) were observed. Moreover, most lower-limb parameters in the sagittal plane had large coefficients of determination without proportional biases, and the ICCs were moderate to excellent. In contrast, although differences in walking speed did not change time–distance ICCs, changes in lower extremity joint angles resulted in less reliable and less valid results. Therefore, OpenPose remains a promising alternative to 3D motion analysis systems, and it can be used in certain gait recognition tasks. As such, future studies should thoroughly examine the cost- and time-reduction potentials of OpenPose for the expected large benefits to society.

## Figures and Tables

**Table 1:** Mean ± SDs of the time–distance factors—Vicon vs. Imasen.

Measurement		Mean Data (L)					Mean Data (R)					Mean Data (LR)					Test–Retest ICC (1,k)			
		Vicon		Imasen		Diff	Vicon		Imasen		Diff	Vicon		Imasen		Diff	Vicon		Imasen	
		Mean	SD	Mean	SD		Mean	SD	Mean	SD		Mean	SD	Mean	SD		L	R	L	R
Step length (mm)	com	699.6	48.7	685.4	47.2	−14.2	696.2	49.4	682.2	48.3	−13.9	697.9	48.4	683.8	47.1	−14.1	0.736	0.906	0.830	0.769
	max	790.5	55.7	780.8	63.6	−9.6	799	51.6	787.7	50.1	−11.2	794.6	53.2	784.2	56.7	−10.4	0.992	0.980	0.983	0.968
	tandem	663.9	77	658.1	78.1	−5.8	663.1	71.3	663.3	69.5	0.2	663.5	73.2	660.7	73	−2.8	0.982	0.932	0.975	0.959
Gait speed (m/s)	com	1.38	0.12	1.36	0.11	−0.02	1.38	0.12	1.37	0.11	−0.02	1.38	0.12	1.37	0.11	−0.02	0.982	0.948	0.977	0.931
	max	1.85	0.18	1.82	0.17	−0.03	1.86	0.17	1.82	0.13	−0.04	1.86	0.17	1.82	0.15	−0.04	0.990	0.992	0.988	0.965
	tandem	1.23	0.23	1.24	0.24	0.01	1.25	0.23	1.24	0.21	0	1.24	0.23	1.24	0.22	0.01	0.977	0.987	0.951	0.987
Stride time (s)	com	1.009	0.046	1.013	0.036	0.005	1.023	0.045	1.021	0.038	−0.002	1.016	0.046	1.017	0.037	0.001	0.921	0.947	0.927	0.955
	max	0.858	0.068	0.869	0.064	0.011	0.874	0.063	0.878	0.059	0.004	0.866	0.065	0.873	0.061	0.008	0.965	0.982	0.947	0.959
	tandem	1.092	0.142	1.099	0.166	0.007	1.086	0.124	1.091	0.125	0.004	1.089	0.131	1.095	0.145	0.005	0.979	0.975	0.981	0.985
Stride length (mm)	com	1385.5	96.6	1377.1	92.6	−8.4	1413	92.5	1393.2	92.4	−19.7	1399.2	94.4	1385.2	91.7	−14	0.949	0.922	0.949	0.911
	max	1580	94.3	1571.2	119.3	−8.7	1615.3	109.8	1589	89.2	−26.3	1597.1	102.3	1579.9	104.6	−17.3	0.992	0.983	0.990	0.978
	tandem	1313.9	141.5	1328.7	146.1	14.8	1327.9	155.5	1332.3	136.1	4.4	1320.9	146.8	1330.5	139.3	9.6	0.969	0.741	0.967	0.958

This table shows the means, SDs, and ICCs of the time factors from the Vicon and Imasen instruments. Abbreviations: com, comfortable gait speed; ICC, intraclass correlation coefficient; max, maximum gait speed; SD, standard deviation; tandem, tandem gait.

**Table 2:** Mean ± SD (degree) of the peak angle and peak phase—Vicon vs. Imasen.

Item																		Test–Retest ICC (1,k)			
			Vicon		Imasen		Diff	Vicon		Imasen		Diff	Vicon		Imasen		Diff	Vicon		Imasen	
			Mean	SD	Mean	SD		Mean	SD	Mean	SD		Mean	SD	Mean	SD		L	R	L	R
TLA	Angle (°)	com	23.6	2.6	22.1	2.4	−1.5	23.6	2.5	23.2	2.1	−0.3	23.6	2.5	22.7	2.3	−0.9	0.978	0.931	0.974	0.815
		max	27.2	2.5	25.6	2.1	−1.6	26.9	2.8	25.9	2.2	−0.9	27	2.6	25.8	2.1	−1.3	0.991	0.975	0.983	0.973
		tandem	22.1	3.2	20.8	2.7	−1.3	22.1	3.3	21.1	3.1	−1	22.1	3.2	21	2.9	−1.2	0.800	0.985	0.844	0.976
Hip joint	Flexion angle (°)	com	31	6.1	27.5	1.9	−3.5	32.5	6.1	32.2	3	−0.3	31.7	6.1	29.8	3.5	−1.9	0.996	0.991	0.954	0.943
		max	33.9	6.4	28.6	2.5	−5.4	34.7	6.1	34.6	2.7	−0.1	34.3	6.2	31.5	4	−2.8	0.993	0.997	0.991	0.941
		tandem	31.6	6.2	27.1	2.6	−4.5	33.2	5.7	32.4	3.8	−0.8	32.4	5.9	29.8	4.2	−2.7	0.961	0.985	0.980	0.915
	Flexion phase (%)	com	13	5.6	6.4	3.8	−6.6	12	4.7	6	2.9	−6	12.5	5.2	6.2	3.3	−6.3	0.941	0.963	0.647	0.885
		max	14.3	6.1	9.3	4	−5	14.7	6.4	9.3	3.3	−5.4	14.5	6.2	9.3	3.7	−5.2	0.979	0.918	0.965	0.829
		tandem	10.4	5.3	5	4	−5.4	10.9	4.8	4.8	2.3	−6.1	10.7	5	4.9	3.2	−5.8	0.994	0.941	0.884	0.880
	Extension angle (%)	com	−14.1	6.7	−12.4	3.1	1.7	−13.3	5.8	−15.3	2.7	−2	−13.7	6.2	−13.8	3.2	−0.1	0.996	0.791	0.983	0.741
		max	−16.1	6.9	−15.7	2.7	0.3	−16.6	6.8	−14.9	3.9	1.7	−16.3	6.8	−15.3	3.3	1	0.995	0.963	0.984	0.886
		tandem	−12.2	8.2	−12.1	3.1	0	−12.5	6.4	−13.1	4.8	−0.7	−12.3	7.3	−12.6	4	−0.3	0.886	0.992	0.846	0.980
	Extension phase (%)	com	70.8	1.8	66.4	1.6	−4.5	71.1	1.4	67.1	1.5	−4	70.9	1.6	66.7	1.6	−4.2	0.962	0.899	0.902	0.763
		max	69.5	2	65.4	1.4	−4.2	69.5	1.8	65.6	1	−3.9	69.5	1.8	65.5	1.3	−4	0.933	0.899	0.754	0.839
		tandem	71.7	2.2	67.5	2.2	−4.2	71.6	2	67.5	1.8	−4.1	71.7	2.1	67.5	2	−4.2	0.979	0.899	0.949	0.871
Knee joint	Flexion angle (°)	com	65.9	8.6	65.2	2.1	−0.7	65.3	11.4	65.3	4.1	0	65.6	9.9	65.3	3.2	−0.3	0.992	0.899	0.927	0.970
		max	66.8	7.6	64.5	3.1	−2.3	63.5	13	69.8	5.6	6.3	65.2	10.6	67.1	5.2	1.9	0.993	0.899	0.983	0.912
		tandem	65.4	7.1	64.3	3.1	−1.1	65.2	10.4	67.7	6.4	2.5	65.3	8.8	66	5.3	0.7	0.993	0.899	0.976	0.961
	Flexion phase (%)	com	90.7	1.3	88.3	1.1	−2.5	91	1.1	89.3	0.9	−1.6	90.9	1.2	88.8	1.1	−2.1	0.989	0.899	0.876	0.626
		max	90.2	1.3	87.6	1	−2.7	90.3	1.3	88.5	1.1	−1.8	90.3	1.3	88	1.2	−2.2	0.979	0.899	0.636	0.756
		tandem	91.7	1.4	88.7	1.1	−3	92	1.1	89.6	1.3	−2.4	91.8	1.2	89.1	1.3	−2.7	0.954	0.899	0.813	0.554
	Extension angle (°)	com	6	7	9.5	3.9	3.5	6.5	7.3	7.7	4.5	1.2	6.2	7.1	8.6	4.3	2.4	0.997	0.899	0.984	0.952
		max	5.7	6.3	9.3	3.8	3.5	3.6	8.7	13.3	6.9	9.7	4.7	7.5	11.2	5.8	6.5	0.995	0.899	0.931	0.909
		tandem	6.4	7.6	8.7	5.5	2.3	5.6	8.5	9.3	7.8	3.6	6	8	9	6.6	3	0.987	0.899	0.930	0.979
	Extension phase (%)	com	57	2.1	54	2	−3	57	2	57.2	1.8	0.2	57	2	55.6	2.5	−1.4	0.914	0.899	0.885	0.927
		max	57.5	1.6	55	1.7	−2.5	57.6	1.7	57.3	1.3	−0.4	57.5	1.6	56.1	1.9	−1.5	0.966	0.899	0.428	0.672
		tandem	54.6	3.9	52.1	4.8	−2.5	54.8	4.1	55.7	3.2	0.9	54.7	4	53.9	4.4	−0.8	0.729	0.899	0.776	0.852

This table shows the means, SDs, and ICCs of the time factors from the Vicon and Imasen instruments. Abbreviations: com, comfortable gait speed; ICC, intraclass correlation coefficient; max, maximum gait speed; SD, standard deviation; tandem, tandem gait; TLA, trailing limb angle; L, Left; R, Right; SD, standard deviation; diff, difference; Imasen, Imasen gait measurement system.

**Table 3:** Regression and ICCs ((3,k)) for gait items—Vicon vs. Imasen.

Item	Gait Pattern	LR	Unstandardized Coefficients B	Constant	95% CI for B (*p* Value)	R^2^	ICC (2, k)	95% CI for ICC (2,k)	Cronbach’s Alpha
Step length	com	L	0.975	**12.33**	**0.809 to 1.142 (*p* = 0.000)**	**0.888**	**0.952**	0.702–0.986	0.972
		R	0.975	**13.312**	**0.820 to 1.130 (*p* = 0.000)**	**0.901**	**0.957**	0.713–0.987	0.975
		LR	0.975	**18.548**	**0.869 to 1.082 (*p* = 0.000)**	**0.898**	**0.953**	0.721–0.984	0.974
	max	L	0.676	**4.997**	**0.391 to 0.962 (*p* = 0.000)**	**0.571**	**0.866**	0.660–0.948	0.867
		R	0.898	**7.144**	**0.632 to 1.165 (*p* = 0.000)**	**0.746**	**0.923**	0.788–0.972	0.932
		LR	0.76	**8.197**	**0.572 to 0.948 (*p* = 0.000)**	**0.648**	**0.888**	0.781–0.943	0.895
	tandem	L	0.933	**12.12**	**0.771 to 1.096 (*p* = 0.000)**	**0.89**	**0.973**	0.930–0.989	0.973
		R	0.993	**15.908**	**0.861 to 1.125 (*p* = 0.000)**	**0.933**	**0.984**	0.960–0.994	0.984
		LR	0.958	**19.39**	**0.858 to 1.058 (*p* = 0.000)**	**0.91**	**0.977**	0.957–0.988	0.977
Gait speed	com	L	1.027	**13.262**	**0.864 to 1.190 (*p* = 0.000)**	**0.902**	**0.971**	0.923–0.989	0.974
		R	1.01	**21.948**	**0.913 to 1.107 (*p* = 0.000)**	**0.962**	**0.986**	0.930–0.995	0.991
		LR	1.019	**23.124**	**0.930 to 1.108 (*p* = 0.000)**	**0.932**	**0.978**	0.943–0.990	0.982
	max	L	0.95	**10.581**	**0.760 to 1.139 (*p* = 0.000)**	**0.86**	**0.957**	0.866–0.984	0.965
		R	1.246	**15.471**	**1.075 to 1.417 (*p* = 0.000)**	**0.933**	**0.953**	0.791–0.985	0.968
		LR	1.057	**16.385**	**0.926 to 1.188 (*p* = 0.000)**	**0.881**	**0.954**	0.859–0.981	0.966
	tandem	L	0.946	**22.341**	**0.857 to 1.036 (*p* = 0.000)**	**0.965**	**0.991**	0.976–0.996	0.991
		R	1.086	**29.946**	**1.009 to 1.162 (*p* = 0.000)**	**0.98**	**0.994**	0.983–0.998	0.993
		LR	1.008	**33.265**	**0.947 to 1.070 (*p* = 0.000)**	**0.968**	**0.992**	0.984–0.996	0.992
Stride time	com	L	1.167	**8.877**	**0.890 to 1.443 (*p* = 0.000)**	**0.804**	**0.932**	0.832–0.973	0.932
		R	1.034	**7.692**	**0.751 to 1.316 (*p* = 0.000)**	**0.754**	**0.93**	0.822–0.972	0.927
		LR	1.105	**11.922**	**0.917 to 1.292 (*p* = 0.000)**	**0.783**	**0.93**	0.869–0.963	0.929
	max	L	0.946	**8.261**	**0.704 to 1.188 (*p* = 0.000)**	**0.789**	**0.94**	0.844–0.977	0.944
		R	1.029	**13.65**	**0.869 to 1.189 (*p* = 0.000)**	**0.916**	**0.978**	0.943–0.992	0.978
		LR	0.987	**14.238**	**0.846 to 1.128 (*p* = 0.000)**	**0.849**	**0.957**	0.914–0.978	0.959
	tandem	L	0.829	**16.527**	**0.723 to 0.935 (*p* = 0.000)**	**0.938**	**0.979**	0.947–0.992	0.979
		R	0.969	**21.006**	**0.872 to 1.066 (*p* = 0.000)**	**0.961**	**0.991**	0.976–0.996	0.991
		LR	0.88	**24.911**	**0.808 to 0.951 (*p* = 0.000)**	**0.944**	**0.983**	0.968–0.991	0.983
Stride length	com	L	0.97	**10.641**	**0.778 to 1.161 (*p* = 0.000)**	**0.855**	**0.963**	0.907–0.985	0.963
		R	0.967	**15.972**	**0.840 to 1.094 (*p* = 0.000)**	**0.93**	**0.973**	0.849–0.992	0.983
		LR	0.974	**18.003**	**0.856 to 1.084 (*p* = 0.000)**	**0.892**	**0.967**	0.927–0.984	0.972
	max	L	0.663	**6.357**	**0.443 to 0.883 (*p* = 0.000)**	**0.686**	**0.902**	0.747–0.963	0.899
		R	1.122	**8.802**	**0.851 to 1.392 (*p* = 0.000)**	**0.818**	**0.928**	0.774–0.975	0.942
		LR	0.83	**9.511**	**0.653 to 1.007 (*p* = 0.000)**	**0.713**	**0.913**	0.830–0.956	0.918
	tandem	L	0.93	**14.475**	**0.795 to 1.066 (*p* = 0.000)**	**0.921**	**0.979**	0.944–0.992	0.98
		R	1.097	**14.144**	**0.934 to 1.261 (*p* = 0.000)**	**0.917**	**0.976**	0.939–0.991	0.975
		LR	1.009	**19.785**	**0.905 to 1.112 (*p* = 0.000)**	**0.913**	**0.977**	0.955–0.988	0.977

Abbreviations: com, comfortable gait speed; max, maximum gait speed; tandem, tandem gait; L, Left; R, Right; ICC, intraclass correlation coefficient. The linear regression analyses were performed using the data obtained by the Imasen gait measurement system as independent variables and the data obtained by Vicon as dependent variables. Only statistically significant variables in regression analysis (*p* values < 0.05) are shown in bold. Moreover, the variables that exceeded thresholds (R^2^ > 0.25, ICC > 0.5) are underlined.

**Table 4:** Regression and ICCs ((3, k)) for peak angles and phases—Vicon vs. Imasen.

Item	Gait Pattern	LR	Unstandardized Coefficients B	Constant	95% CI for B (*p* Value)	R^2^	ICC (3, k)	95% CI for ICC (3, k)	Cronbach’s Alpha
TLA	com	L	1.052	**11.333**	**0.857 to 1.247 (*p* = 0.000)**	**0.87**	**0.964**	0.909–0.986	0.964
		R	0.977	**6.209**	**0.646 to 1.308 (*p* = 0.000)**	**0.664**	**0.898**	0.741–0.959	0.898
		LR	0.948	**10.034**	**0.757 to 1.139 (*p* = 0.000)**	**0.719**	**0.917**	0.844–0.956	0.917
	max	L	1.122	**10.19**	**0.890 to 1.355 (*p* = 0.000)**	**0.851**	**0.953**	0.878–0.982	0.953
		R	1.067	**6.382**	**0.713 to 1.422 (*p* = 0.000)**	**0.7**	**0.904**	0.744–0.964	0.904
		LR	1.081	**10.693**	**0.876 to 1.286 (*p* = 0.000)**	**0.759**	**0.922**	0.849–0.960	0.922
	tandem	L	1.144	**13.782**	**0.969 to 1.319 (*p* = 0.000)**	**0.913**	**0.971**	0.924–0.989	0.971
		R	0.994	**10.65**	**0.797 to 1.191 (*p* = 0.000)**	**0.862**	**0.964**	0.907–0.986	0.964
		LR	1.055	**16.664**	**0.927 to 1.184 (*p* = 0.000)**	**0.882**	**0.966**	0.935–0.982	0.966
Hip									
com	flexion	L	1.018	2.562	0.183 to 1.854 (*p* = 0.200)	0.226	**0.589**	−0.039–0.837	0.589
		R	0.737	2.449	0.105 to 1.369 (*p* = 0.250)	0.208	**0.634**	0.076–0.855	0.634
		LR	0.854	**3.596**	**0.373 to 1.335 (*p* = 0.000)**	0.234	**0.612**	0.266–0.795	0.301
	extension	L	1.39	**7.204**	**0.985 to 1.796 (*p* = 0.000)**	**0.728**	**0.871**	0.674–0.949	0.871
		R	1.013	**4.843**	**0.573 to 1.452 (*p* = 0.000)**	**0.542**	**0.837**	0.589–0.936	0.837
		LR	1.186	**8.318**	**0.897 to 1.474 (*p* = 0.000)**	**0.636**	**0.855**	0.725–0.923	0.855
max	flexion	L	0.267	0.783	−0.452 to 0.986 (*p* = 0.445)	0.022	0.298	−0.822–0.730	0.298
		R	0.371	1.021	−0.400 to 1.142 (*p* = 0.322)	0.02	0.372	−0.680–0.765	0.372
		LR	0.318	1.316	−0.172 to 0.808 (*p* = 0.197)	0.02	0.336	−0.289–0.658	0.336
	extension	L	1.596	**7.308**	**1.136 to 2.057 (*p* = 0.000)**	**0.744**	**0.845**	0.599–0.940	0.845
		R	1.231	**5.129**	**0.722 to 1.739 (*p* = 0.000)**	**0.598**	**0.835**	0.558–0.938	0.835
		LR	1.397	**8.731**	**1.072 to 1.722 (*p* = 0.000)**	**0.676**	**0.841**	0.692–0.918	0.841
tandem	flexion	L	0.663	**2.187**	**0.023 to 1.302 (*p* = 0.043)**	0.174	**0.613**	−0.005–0.851	0.613
		R	0.865	**2.747**	**0.201 to 1.530 (*p* = 0.014)**	**0.267**	**0.67**	0.144–0.873	0.67
		LR	0.75	**3.568**	**0.324 to 1.176 (*p* = 0.001)**	0.241	**0.645**	0.316–0.815	0.645
	extension	L	1.657	**7.455**	**1.188 to 2.126 (*p* = 0.000)**	**0.752**	**0.839**	0.582–0.938	0.839
		R	1.019	**5.803**	**0.176 to 0.815 (*p* = 0.000)**	**0.645**	**0.886**	0.704–0.956	0.886
		LR	1.246	**8.775**	**0.142 to 0.825 (*p* = 0.000)**	**0.673**	**0.864**	0.738–0.929	0.864
knee									
com	flexion	L	2.226	**3.652**	**0.945 to 3.507 (*p* = 0.002)**	**0.394**	**0.521**	−0.211–0.810	0.521
		R	2.241	**4.43**	**1.178 to 3.303 (*p* = 0.000)**	**0.495**	**0.593**	−0.027–0.839	0.593
		LR	2.228	**6.008**	**1.477 to 2.979 (*p* = 0.000)**	**0.474**	**0.57**	0.186–0.772	0.57
	extension	L	1.129	**3.579**	**0.466 to 1.791 (*p* = 0.002)**	**0.383**	**0.714**	0.278–0.887	0.714
		R	1.174	**3.452**	**0.460 to 1.889 (*p* = 0.003)**	**0.365**	**0.69**	0.216–0.87	0.69
		LR	1.125	**5.044**	**0.674 to 1.577 (*p* = 0.000)**	**0.385**	**0.702**	0.437–0.843	0.702
max	flexion	L	1.119	**2.625**	**0.220 to 2.019 (*p* = 0.018)**	0.247	**0.591**	−0.063–0.842	0.591
		R	2.095	**3.425**	**0.798 to 3.391 (*p* = 0.003)**	**0.387**	**0.538**	−0.234–0.827	0.538
		LR	1.692	**4.388**	**0.909 to 2.475 (*p* = 0.000)**	**0.336**	**0.544**	0.113–0.765	0.544
	extension	L	0.821	**2.429**	**0.108 to 1.534 (*p* = 0.027)**	0.214	**0.625**	0.025–0.855	0.625
		R	1.624	**4.67**	**0.887 to 2.361 (*p* = 0.000)**	**0.55**	**0.737**	0.296–0.901	0.737
		LR	1.172	**4.629**	**0.658 to 1.686 (*p* = 0.000)**	**0.362**	**0.673**	0.366–0.832	0.673
tandem	flexion	L	1.229	**2.794**	**0.301 to 2.156 (*p* = 0.012)**	**0.274**	**0.595**	−0.050–0.844	0.595
		R	1.697	**4.017**	**0.806 to 2.589 (*p* = 0.000)**	**0.457**	**0.659**	0.114–0.868	0.659
		LR	1.51	**5.088**	**0.908 to 2.112 (*p* = 0.000)**	**0.402**	**0.638**	0.303–0.812	0.638
	extension	L	0.83	**3.249**	**0.291 to 1.369 (*p* = 0.005)**	**0.347**	**0.745**	0.337–0.902	0.745
		R	1.054	**4.235**	**0.529 to 1.579 (*p* = 0.000)**	**0.485**	**0.8**	0.480–0.923	0.8
		LR	0.929	**5.298**	**0.574 to 1.285 (*p* = 0.000)**	**0.423**	**0.77**	0.557–0.880	0.77

Abbreviations: com, comfortable gait speed; max, maximum gait speed; tandem, tandem gait; L, Left; R, Right; ICC, intraclass correlation coefficient; CI, confidence interval; TLA, trailing limb angle. The linear regression analyses were performed by using the data obtained by the Imasen gait measurement system as independent variables and the data obtained by Vicon as dependent variables. Only statistically significant variables in regression analysis (*p* values < 0.05) are shown in bold. Moreover, the variables that exceeded thresholds (R^2^ > 0.25, ICC > 0.5) are underlined.

**Table 5:** Bland–Altman plots: fixed and proportional biases for time–distance factors—Vicon vs. Imasen system.

Parameter	Gait Pattern	LR	Fixed Bias			Proportional Bias
			Bias	95% CI for Bias (*p* Value)	LoA (Upper–Lower)	Regression Equation (*p* Value)
Step length	com	L	14.215	**6.787 to 21.643 (*p* = 0.001)**	−16.895 to 45.325	y = 0.032x − 7.915 (*p* = 0.690)
		R	13.92	**6.827 to 21.013 (*p* = 0.001)**	−15.785 to 43.625	y = 0.024x − 2.687 (*p* = 0.748)
		LR	14.068	**9.168 to 18.967 (*p* = 0.000)**	−15.957 to 44.092	y = 0.028x − 5.292 (*p* = 0.597)
	max	L	9.647	−10.120 to 29.415 (*p* = 0.319)	−70.737 to 90.031	y = −0.148x + 125.974 (*p* = 0.405)
		R	11.25	−1.528 to 24.028 (*p* = 0.081)	−39.114 to 61.614	y = 0.031x − 13.402 (*p* = 0.815)
		LR	10.427	−0.897 to 21.751 (*p* = 0.070)	−56.140 to 76.994	y = −0.071x + 66.694 (*p* = 0.518)
	tandem	L	5.816	−6.400 to 18.032 (*p* = 0.330)	−43.860 to 55.491	y =−0.015x + 15.465 (*p* = 0.858)
		R	−0.205	−8.833 to 8.422 (*p* = 0.961)	−35.289 to 34.879	y = 0.026x − 17.359 (*p* = 0.681)
		LR	2.805	−4.379 to 9.989 (*p* = 0.434)	−40.032 to 45.643	y = 0.003x + 0.723 (*p* = 0.951)
Gait speed	com	L	0.016	−0.002 to 0.034 (*p* = 0.080)	−0.060 to 0.092	y = 0.084x − 0.099 (*p* = 0.281)
		R	0.017	**0.006 to 0.027 (*p* = 0.005)**	−0.029 to 0.062	y = 0.040x − 0.039 (*p* = 0.399)
		LR	0.016	**0.006 to 0.026 (*p* = 0.002)**	−0.045 to 0.078	y = 0.063x − 0.070 (*p* = 0.158)
	max	L	0.036	**0.005 to 0.068 (*p* = 0.025)**	−0.091 to 0.163	y = 0.019x + 0.002 (*p* = 0.842)
		R	0.041	**0.014 to 0.067 (*p* = 0.005)**	−0.063 to 0.144	**y = 0.248x − 0.415 (*p* = 0.001)**
		LR	0.038	**0.019 to 0.058 (*p* = 0.000)**	−0.076 to 0.153	y = 0.116x − 0.175 (*p* = 0.055)
	tandem	L	−0.013	−0.035 to 0.007 (*p* = 0.186)	−0.099 to 0.071	y = −0.037x + 0.032 (*p* = 0.425)
		R	0.001	−0.016 to 0.017 (*p* = 0.948)	−0.067 to 0.068	y = 0.091x − 0.113 (*p* = 0.009)
		LR	−0.007	−0.020 to 0.006 (*p* = 0.309)	−0.084 to 0.071	y = 0.025x − 0.038 (*p* = 0.393)
Stride time	com	L	−0.005	−0.015 to 0.005 (*p* = 0.322)	−0.046 to 0.036	**y = 0.268x − 0.276 (*p* = 0.020)**
		R	0.002	−0.008 to 0.012 (*p* = 0.666)	−0.041 to 0.045	y = 0.177x − 0.178 (*p* = 0.160)
		LR	−0.001	−0.008 to 0.006 (*p* = 0.703)	−0.043 to 0.041	**y = 0.230x − 0.235 (*p* = 0.005)**
	max	L	−0.011	−0.026 to 0.004 (*p* = 0.139)	−0.071 to 0.049	y = 0.059x − 0.062 (*p* = 0.613)
		R	−0.004	−0.013 to 0.005 (*p* = 0.329)	−0.039 to 0.031	y = 0.071x − 0.067 (*p* = 0.336)
		LR	−0.008	−0.016 to 0.001 (*p* = 0.072)	−0.057 to 0.041	y = 0.069x − 0.068 (*p* = 0.310)
	tandem	L	−0.007	−0.028 to 0.015 (*p* = 0.531)	−0.094 to 0.081	**y = −0.159x + 0.168 (*p* = 0.015)**
		R	−0.004	−0.016 to 0.007 (*p* = 0.441)	−0.052 to 0.043	y = − 0.013x + 0.009 (*p* = 0.792)
		LR	−0.005	−0.017 to 0.006 (*p* = 0.348)	−0.075 to 0.064	**y = −0.101x + 0.105 (*p* = 0.015)**
Stride length	com	L	8.2	−8.593 to 24.993 (*p* = 0.320)	−62.126 to 78.526	y = 0.044x − 53.201 (*p* = 0.629)
		R	19.65	**8.450 to 30.850 (*p* = 0.002)**	−27.253 to 66.553	y = 0.001x + 18.656 (*p* = 0.991)
		LR	13.925	**4.121 to 23.729 (*p* = 0.007)**	−46.162 to 74.012	y = 0.030x − 28.004 (*p* = 0.580)
	max	L	8.632	−22.800 to 40.063 (*p* = 0.571)	−119.184 to 136.447	y = −0.255x + 410.081 (*p* = 0.089)
		R	26.222	3.001 to 49.443 (*p* = 0.029)	−65.300 to 117.744	y = 0.217x − 322.206 (*p* = 0.059)
		LR	−0.877	**−0.898 to −0.856 (*p* = 0.000)**	−1.000 to −0.754	**y = −1.284x − 0.324 (*p* = 0.000)**
	tandem	L	−14.895	−34.194 to 4.405 (*p* = 0.122)	−93.376 to 63.587	y = −0.034x + 29.652 (*p* = 0.625)
		R	−4.368	−26.311 to 17.572 (*p* = 0.681)	−93.599 to 84.862	y = 0.136x − 185.575 (*p* = 0.065)
		LR	−9.632	−23.641 to 4.378 (*p* = 0.172)	−93.173 to 73.910	y = 0.054x − 80.695 (*p* = 0.285)

Abbreviations: com, comfortable gait speed; max, maximum gait speed; tandem, tandem gait; Imasen, Imasen gait measurement system; CI, confidence intervals. Denotes difference between the data obtained by Imasen and Vicon systems. Only statistically significant variables (*p* values < 0.05) are shown in bold.

**Table 6:** Bland–Altman plots: fixed and proportional biases for peak angles and ranges—Vicon vs. Imasen system.

			Fixed Bias			Proportional Bias
			Bias	95% CI for Bias (*p* Value)	LoA (Upper–Lower)	Regression Equation (*p* Value)
TLA	com	L	1.515	**1.088 to 1.942 (*p* = 0.000)**	−0.273 to 3.303	y = 0.119x − 1.199 (*p* = 0.169)
		R	0.315	−0.348 to 0.978 (*p* = 0.332)	−2.461 to 3.091	y = 0.184x − 3.989 (*p* = 0.220)
		LR	0.915	**0.492 to 1.338 (*p* = 0.000)**	−1.679 to 3.509	y = 0.115x − 1.738 (*p* = 0.213)
	max	L	1.574	**1.098 to 2.049 (*p* = 0.000)**	−0.361 to 3.508	**y = 0.204x − 3.824 (*p* = 0.045)**
		R	0.917	**0.170 to 1.663 (*p* = 0.019)**	−2.025 to 3.858	**y = 0.204x − 3.824 (*p* = 0.045)**
		LR	1.254	**0.824 to 1.683 (*p* = 0.000)**	−1.272 to 3.780	**y = 0.226x − 4.709 (*p* = 0.014)**
	tandem	L	1.342	**0.858 to 1.827 (*p* = 0.000)**	−0.628 to 3.312	**y = 0.181x − 2.537 (*p* = 0.020)**
		R	0.989	**0.418 to 1.561 (*p* = 0.002)**	−1.333 to 3.312	y = 0.063x − 0.374 (*p* = 0.492)
		LR	1.166	**0.805 to 1.527 (*p* = 0.000)**	−0.987 to 3.319	y = 0.117x − 1.348 (*p* = 0.050)
Hip						
com	flexion	L	3.48	0.718 to 6.242 (*p* = 0.016)	−8.086 to 15.046	**y = 1.444x − 38.717 (*p* = 0.000)**
		R	0.29	−2.762 to 3.342 (*p* = 0.844)	−12.490 to 13.070	**y = 1.103x − 35.362 (*p* = 0.005)**
		LR	1.885	−0.145 to 3.915 (*p* = 0.068)	−10.556 to 14.326	**y = 0.873x − 24.963 (*p* = 0.001)**
	extension	L	−1.695	−4.487 to 1.097 (*p* = 0.219)	−13.387 to 9.997	**y = 0.961x + 10.987 (*p* = 0.001)**
		**R**	**1.98**	**−0.924 to 4.884 (*p* = 0.000)**	−10.180 to 14.140	**y = 1.187x + 18.913 (*p* = 0.002)**
		**LR**	**0.143**	**−1.869 to 2.154 (*p* = 0.000)**	−12.184 to 12.469	**y = 0.958x + 13.301 (*p* = 0.000)**
max	flexion	**L**	**5.347**	**2.085 to 8.610 (*p* = 0.003)**	−7.920 to 18.614	**y = 1.462x − 40.308 (*p* = 0.000)**
		R	0.089	−3.407 to 3.585 (*p* = 0.958)	−13.691 to 13.869	**y = 1.479x − 51.210 (*p* = 0.002)**
		**LR**	**2.789**	**0.354 to 5.224 (*p* = 0.026)**	−11.527 to 17.105	**y = 0.800x − 23.526 (*p* = 0.013)**
	extension	L	−0.337	−3.357 to 2.683 (*p* = 0.817)	−12.619 to 11.945	**y = 1.154x + 18.004 (*p* = 0.000)**
		R	0.089	−3.407 to 3.585 (*p* = 0.958)	−13.691 to 13.869	**y = 1.479x − 51.210 (*p* = 0.002)**
		LR	−1.008	−3.222 to 1.206 (*p* = 0.362)	−14.022 to 12.006	**y = 1.011x + 15.016 (*p* = 0.000)**
tandem	flexion	**L**	**4.463**	**1.305 to 7.621 (*p* = 0.008)**	−8.379 to 17.305	**y = 1.328x − 34.560 (*p* = 0.001)**
		R	0.837	−2.603 to 4.276 (*p* = 0.615)	−13.150 to 14.823	y = 0.825x − 26.217 (*p* = 0.108)
		**LR**	**−2.95**	**−5.412 to −0.488 (*p* = 0.020)**	−17.629 to 11.729	y = 0.244x − 4.783 (*p* = 0.215)
	extension	L	−0.037	−3.296 to 3.222 (*p* = 0.981)	−13.291 to 13.217	**y = 1.066x + 12.899 (*p* = 0.000)**
		R	0.658	−2.566 to 3.882 (*p* = 0.673)	−12.451 to 13.767	y = 0.427x + 6.129 (*p* = 0.221)
		LR	0.311	−1.873 to 2.494 (*p* = 0.775)	−12.710 to 13.331	**y = 0.776x + 9.985 (*p* = 0.000)**
Knee						
com	flexion	L	0.71	−2.695 to 4.115 (*p* = 0.667)	−13.550 to 14.970	**y = 1.339x − 87.008 (*p* = 0.000)**
		R	−0.035	−4.716 to 4.646 (*p* = 0.988)	−19.639 to 19.569	**y = 1.174x − 76.726 (*p* = 0.000)**
		LR	0.338	−2.426 to 3.101 (*p* = 0.806)	−16.599 to 17.274	**y = 1.232x − 80.288 (*p* = 0.000)**
	extension	L	−3.53	**−6.060 to 1.000 (*p* = 0.009)**	−14.123 to 7.063	**y = 0.679x − 8.795 (*p* = 0.003)**
		R	−1.22	−4.769 to 2.329 (*p* = 0.481)	−16.083 to 13.643	**y = 0.746x − 6.505 (*p* = 0.038)**
		LR	−2.375	**−4.487 to −0.263 (*p* = 0.029)**	−15.319 to 10.569	**y = 0.690x − 7.495 (*p* = 0.001)**
max	flexion	L	2.311	−0.828 to 5.449 (*p* = 0.139)	−10.451 to 15.072	**y = 1.040x − 65.994 (*p* = 0.000)**
		R	−6.317	−13.728 to 1.095 (*p* = 0.090)	−35.528 to 22.895	**y = 1.539x − 109.002 (*p* = 0.001)**
		LR	−1.886	−5.904 to 2.131 (*p* = 0.347)	−0.725 to 4.951	**y = 1.287x − 87.037 (*p* = 0.000)**
	extension	L	−3.521	**−6.160 to −0.882 (*p* = 0.012)**	−14.252 to 7.210	**y = 0.665x − 8.515 (*p* = 0.017)**
		R	−9.739	**−15.514 to −3.963 (*p* = 0.002)**	−32.502 to 13.025	y = 0.481x − 13.804 (*p* = 0.382)
		LR	−6.546	**−9.685 to −3.407 (*p* = 0.000)**	−24.996 to 11.904	y = 0.490x − 10.446 (*p* = 0.134)
tandem	flexion	L	1.089	−1.922 to 34.101 (*p* = 0.457)	−11.158 to 13.337	**y = 1.007x − 64.210 (*p* = 0.000)**
		R	−2.474	−6.716 to 1.768 (*p* = 0.236)	−19.724 to 14.777	**y = 0.599x − 42.236 (*p* = 0.027)**
		LR	−0.692	−3.237 to 1.853 (*p* = 0.585)	−15.867 to 14.482	**y = 0.659x − 43.948 (*p* = 0.001)**
	extension	L	−2.289	**−5.220 to 0.641 (*p* = 0.001)**	−14.208 to 9.629	y = 0.398x − 5.309 (*p* = 0.103)
		R	−3.611	−7.851 to 0.630 (*p* = 0.090)	−20.853 to 13.632	y = 0.129 − 4.573 (*p* = 0.680)
		LR	−2.95	**−5.412 to −0.488 (*p* = 0.020)**	−17.629 to 11.729	y = 0.244 − 4.783 (*p* = 0.215)

Abbreviations: com, comfortable gait speed; max, maximum gait speed; tandem, tandem gait; Imasen, Imasen gait measurement system; CI, confidence interval; TLA, trailing limb angle. Denotes difference between the data obtained by Imasen and Vicon systems. Only statistically significant variables (*p* values < 0.05) are shown in bold.

## Data Availability

The data presented in this study are available upon request from the corresponding author. The data are not publicly available due to ethical restrictions.
